# Effect of ABCB1 polymorphism on the clinical outcome of osteosarcoma patients after receiving chemotherapy

**DOI:** 10.12669/pjms.304.4955

**Published:** 2014

**Authors:** Sun Xiaohui, Li Aiguo, Geng Xiaolin, Liu Ying, Zhao Hongxing, Zhao Yilei

**Affiliations:** 1 Sun Xiaohui, Department of Orthopedic Surgery, The first affiliated Hospital of Xinxiang Medical University, Weihui, Henan, China.; 2 Li Aiguo, Department of Orthopedic Surgery, The first affiliated Hospital of Xinxiang Medical University, Weihui, Henan, China.; 3 Geng Xiaolin, Department of Orthopedic Surgery, The first affiliated Hospital of Xinxiang Medical University, Weihui, Henan, China.; 4 Liu Ying, Department of Obstetrics and Gynecology, The first affiliated Hospital of Xinxiang Medical University, Weihui, Henan, China.; 5 Zhao Hongxing, Department of Orthopedic Surgery, The first affiliated Hospital of Xinxiang Medical University, Weihui, Henan, China.; 6 Zhao Yilei, Department of Orthopedic Surgery, The first affiliated Hospital of Xinxiang Medical University, Weihui, Henan, China.

**Keywords:** APT-binding cassette, Chemotherapy, Clinical outcome, Osteosarcoma

## Abstract

***Objective: ***To investigate the role of three genetic polymorphisms of ABC proteins in response to chemotherapy and overall survival of osteosarcoma patients.

***Methods: *** A prospective study was conducted. Genotyping analyses of ABCB1 C3435T, ABCG2 C421A, and ABCC3 C-211T were conducted using the TaqMan methodology. Multivariate Cox proportional hazards models were used to calculate hazard ratio (HR) and 95% CI of effect of each genotype of ABCB1 C3435T, ABCG2 c421A, and ABCC3 C-211T on PFS and OS.

***Results:*** During the follow-up period, 135 patients (74.18%) were alive and 47 died (25.82). The median follow-up periods were 36.7 months. Individuals carrying with ABCB1 3435TT genotype and T allele showed less likely to have a poor response to chemotherapy. Cox regression analysis showed that individuals with ABCB1 TT genotype and T allele were associated with high risk of death from osteosarcoma when compared with wide-type genotype. However, we did not find significant association between ABCG2 C421A and ABCC3 C-211T polymorphisms and overall survival of osteosarcoma.

***Conclusion:*** ABCB1 C3435T polymorphism may be used as a genetic predictor of clinical outcome in osteosarcoma patients treated with chemotherapy. However, no association was found between polymorphisms in ABCG2 C421A and ABCC3 C-211T and clinical outcome of osteosarcoma.

## INTRODUCTION

Osteosarcoma is the most common primary malignant bone tumor worldwide, and it accounts for about 20% of all primary sarcomas in bone.^1^ It is estimated that the incidence of osteosarcoma in youths less than 20 years old is about 8.7 per 10 populations, and osteosarcoma is a leading cause of death of these youths.^2^^,^^3^ Although advanced therapies with aggressive adjuvant chemotherapy and wide tumor resection could improve the overall survival of osteosarcoma,^4^ patients with metastatic disease always present a shorter survival time.^5^ It is estimated that about 40% of patients with osteosarcoma show a poor response to chemotherapy, and the five-year survival rate is less than 50%.^6^ However, patients with same tumor stages show different response to chemotherapy and different survival time. Therefore, genetic factors may influence chemotherapy toxicity and clinical outcome of osteosarcoma.^7^^-^^9^

It is suggested that individualized chemotherapy according to some biomarkers can influence the clinical response to chemotherapeutics and prolong the life of patients with osteosarcoma. The ATP-binding cassette (ABC proteins) transports superfamilies and is responsible for the majority of drug transport.^10^ It is reported that many drug metabolizers and transporters present genetic variations, which could change the functional expression of the ABC proteins, and thus influence efficacy of chemotherapeutic agents and prognosis of diseases.

We previously reported a study between GSTs polymorphisms and prognosis of osteosarcoma patients treated with chemotherapy,^8^ and found that the genetic variations could influence the pharmacokinetics and pharmacodynamics of chemotherapeutic drugs. In this study, we planned to investigate the role of three genetic polymorphisms (ABCB1 C3435T, ABCG2 c421A, and ABCC3 C-211T) in response to chemotherapy and overall survival of osteosarcoma patients.

## METHODS

***Subjects***: The study population comprised 182 diagnosed osteosarcoma patients between September 2007 and September 2009 at the first affiliated hospital of Xinxiang Medical University. All cases were newly and histologically diagnosed with primary osteosarcoma. All cases signed an informed consent Form before participating in the study which was approved by the ethics committee of the first affiliated hospital of Xinxiang Medical University.

Osteosarcoma patients received chemotherapy before operation, and the chemotherapy treatment was described in our previous study.^8^ The treatment response to chemotherapy was classified according to histological extent of tumor necrosis. Poor responders were defined as less than 90% of tumor necrosis, and good responders were defined as 90% of tumor necrosis or more.^11^ All the patients were followed up until 31^th^ September 2012, with a median follow-up period of 41.5 months (ranged from 2 to 60 months). All patients were followed up by telephone or outpatient service every two months until death or the end of study (31^th^ September 2012). Overall survival (OS) was used as the end point, and the overall survival was calculated from the date of enrolling in this study to the date of death from any cause or last known date alive or last clinical follow-up.

***DNA extraction and Genotyping*** *: *All study participants were asked to provide 5 ml of peripheral venous blood, and the blood samples were kept at -20ºC. According to the manufacturer’s instructions, genomic DNA was extracted from peripheral venous blood samples with the TIANamp blood DNA kit (Tiangen Biotech, Beijing, China). Genotyping analyses of ABCB1 C3435T, ABCG2 C421A, and ABCC3 C-211T were conducted using the TaqMan methodology in a 384-well plate format on the Sequenom MassARRAY platform and read by Sequence Detection software on the ABI prism 7900 instrument (Sequenom, San Diego, USA), which described in previous study.^8^ The primers used for ABCB1 C3435T, ABCG2 c421A, and ABCC3 C-211T were designed using Sequenom Assay Design 3.1 software (Sequenom®) according to the manufacturer instructions (Table-I). About 10% randomly chosen subgroup were selected from cases and control subjects, and the results of repeated samples were 100% concordant.

**Table-I:** Primers used for ABCB1 C3435T, ABCG2 c421A, and ABCC3 C-211T polymorphisms

***Polymorphism***	***Forward primer sequence (5’-3’)***	***Reverse primer sequence (5’-3’)***
ABCB1 C3435T	TGCTGGTCCTGAAGTTGATCTGTGAAC	ACATTAGGCAGTGACTCGATGAAGGCA
ABCG2 c421A	AATGTATTGTCACCTAGTGTTTG	AGTAAATGCCTTCAGGTCAT
ABCC3 C-211T	GACTCGACTCCTGGACTGTTAGC	CCCTACCCCAGTGCCTCT

***Statistical analysis*** *: *Association between tumor response and genotypes was assessed using logistic regression analysis. Odds ratios (OR) and corresponding 95% confidence intervals (CI) were calculated by comparing genotypes frequencies in good responders versus poor responders. Homozygotes for the most frequent genotype were used as the reference group. Multivariate Cox proportional hazards models were used to calculate hazard ratio (HR) and 95% CI of effect of ABCB1 C3435T, ABCG2 c421A, and ABCC3 C-211T polymorphisms on PFS and OS with or without adjustment for confounding factors. Overall survival times were plotted using Kaplan-Meier product limit method, and the differences between them were estimated by log-rank test. All *P* values were two-tailed, and *P*<0.05 was considered as statistically significant, and the SPSS® statistical package, version 11.0 (SPSS Inc., Chicago, IL, USA) for Windows® were used for statistical analyses. 

## RESULTS

A total of 182 patients with osteosarcoma were enrolled in this study. Table-II shows the clinical characteristics of cases. The mean age of the 182 patients were 16.7 years old (ranged from 9.1 to 58.6 years old), and there were 105 males (57.69%) and 77 females (42.31%). The tumors of 86 patients were at femur (47.25%), and 64 were at tibia/fibula (35.16%). Total 87 patients showed good response to chemotherapy (47.80%), and 95 showed poor response (52.20%). 

Forty patients showed metastasis during the follow-up period, and 45 patients presented metastasis at the time of diagnosis. During the follow-up period, 135 patients (74.18%) were alive and 47 died (25.82%). The median follow-up periods were 36.7 months.

**Table-II:** Clinical and pathological characteristics of included study

***Age at diagnosis, y***	***Patients, N***	***%***
Total number of patients	182	
Median (range)	16.7 (9.1-58.6)	
≤15	79	43.4
>15	103	56.6
Sex		
Male	105	57.7
Female	77	42.3
Tumor location		
Femur	86	47.3
Tibia/fibula	64	35.2
Arm	14	7.7
Central	18	9.9
Necrosis		
Good	95	52.2
Poor	87	47.8
Metastasis		
Absent	97	53.3
At diagnosis	45	24.7
At follow up	40	22.0
Death		
No	125	68.7
Yes	57	31.3

Our results indicated that individuals carrying with ABCB1 3435TT genotype and T allele showed were less likely to have a poor response to chemotherapy, with ORs(95%CI) of 0.38(0.15-0.94) and 0.57(0.37-0.90), respectively (Table-III). However, individuals with ABCG2 C421A and ABCC3 C-211T polymorphisms had no association with response to chemotherapy in patients with osteosarcoma.

**Table-III:** Gene polymorphisms association with tumor response to chemotherapy

***Genotype***		***Patients***	***Tumor response***					***OR(95%CI)*** ^1^	***P value***
			***%***	***Poor***	***%***	***Good***	***%***		
ABCB1 C3435T	CC	77	42.31	31	35.2	47	49.7	1.0(Ref.)	-
	CT	68	37.36	34	39.5	35	37.1	0.65(0.32-1.31)	0.19
	TT	37	20.33	22	25.3	13	13.2	0.38(0.15-0.94)	0.02
	C allele	222	60.99	96	55.0	130	68.3	1.0(Ref.)	-
	T allele	142	39.01	78	45.1	60	31.8	0.57(0.37-0.90)	0.01
ABCG2 C421A	CC	132	72.53	54	62.1	62	65.2	1.0(Ref.)	-
	CA	44	24.18	20	23.2	21	22.1	0.90(0.42-1.91)	0.77
	AA	6	3.30	13	14.7	12	12.7	0.75(0.24-2.22)	0.57
	A allele	308	84.62	128	73.7	145	76.3	1.0(Ref.)	-
	G allele	56	15.38	46	26.3	45	23.8	0.84(0.50-1.42)	0.49
ABCC3 C-211T	CC	85	46.70	38	44.2	46	48.2	1.0(Ref.)	-
	CT	64	35.16	30	34.9	31	32.5	0.86(0.43-1.73)	0.65
	TT	33	18.13	18	20.9	18	19.3	0.71(0.28-1.74)	0.41
	C allele	234	64.29	107	123.3	122	128.9	1.0(Ref.)	-
	T allele	130	35.71	67	76.7	68	71.1	0.81(0.51-1.28)	0.35

1. Adjusted for sex, age, tumor location and metastasis

**Fig.1:**
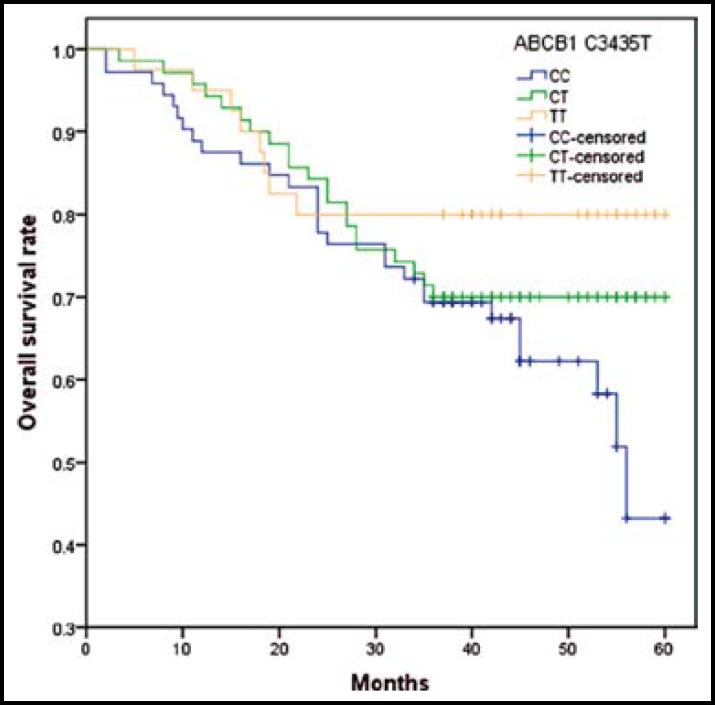
Kaplan-Meier survival curve of ABCB1 C3435T polymorphisms

Cox regression analysis of association between ABCB1 C3435T, ABCG2 C421A and ABCC3 C-211T polymorphisms and the survival of osteosarcoma were showed in Table-IV. Log-rank test showed that individuals carrying ABCB1 TT genotype and T allele had longer survival times than CC genotype (Fig.1). The results showed that individuals with ABCB1 TT genotype and T allele were associated with high risk of death from osteosarcoma when compared with wide-type genotype, with HRs(95%CI) of 2.58(1.03-7.28) and 1.65(1.03-2.72), respectively. However, we did not find significant association between ABCG2 C421A and ABCC3 C-211T polymorphisms and overall survival of osteosarcoma.

**Table-IV:** Cox regression analysis of ABCB1 C3435T, ABCG2 C421A and ABCC3 C-211T polymorphisms with the survival of osteosarcoma

***Genotype***		***Months***	***P value for log-rank test***	***Overall survival***				***HR (95%CI)***	***P value***
				***Death***	***%***	***Alive***	***%***		
ABCB1 C3435T	CC	35.3		20	35.1	62	49.6	1.0(Ref.)	-
	CT	41.4		23	40.4	46	36.8	1.48(0.70-3.17)	0.24
	TT	48.6	0.03	14	24.6	17	13.6	2.58(1.03-7.28)	0.04
	C allele	39.5		63	110.5	170	136	1.0(Ref.)	-
	T allele	46.9	0.02	51	89.5	80	64	1.65(1.03-2.72)	0.03
ABCG2 C421A	CC	38.6		35	61.4	82	65.6	1.0(Ref.)	-
	CA	42.1		13	22.8	27	21.6	1.05(0.47-2.49)	0.83
	AA	44.7	0.51	8	14	16	12.8	1.22(0.44-3.77)	0.65
	A allele	39.4		83	145.6	191	152.8	1.0(Ref.)	-
	G allele	43.2	0.42	29	50.9	59	47.2	1.16(0.67-2.02)	0.58
ABCC3 C-211T	CC	37.1		25	43.9	60	48	1.0(Ref.)	-
	CT	42.3		20	35.1	41	32.8	1.14(0.53-2.46)	0.72
	TT	43.5	0.63	12	21.1	24	19.2	1.16(0.47-3.02)	0.73
	C allele	39.1		70	122.8	161	128.8	1.0(Ref.)	-
	T allele	42.7	0.48	44	77.2	89	71.2	1.11(0.68-1.81)	0.65

## DISCUSSION

In this study, we found that common genetic polymorphisms in APT-binding cassette were associated with increased risk of overall survival in a Chinese population. Although the chemotherapy is selected based on the histological type and tumor stage of osteosarcoma patients, genotyping of genomic DNA could guide the therapy and improve efficacy of treatment. Therefore, we investigated that polymorphisms in ABCB1, ABCG2 and ABCC3 could be a predictor for treatment response for osteosarcoma patients.

It is reported that ABCB1 C3435T gene has a role in encoding a plasma membrane pump, P-glycoprotein, and this gene can protect the cells and organs from damages of xenobiotic agents and environmental carcinogens through draining various structurally unrelated anticancer agents and toxins.^12^^,^^13^ Previous studies have indicated that overall expression of ATP binding cassette transporter, such as ABCB1, ABCG2 and ABCC3, has a direct role in resistance to a broad spectrum of chemotherapeutic agents in vitro, such as anthracyclines, paclitaxel and Vinca alkaloids.^14^ ABCB1 polymorphism can be the one of the main factors in limiting the efficacy of chemotherapeutic agents in several types of cancerr.^15^^-^^17^ Previous three studies have reported the association between ABCB1 polymorphism and response to chemotherapy in patients with osteosarcoma.^18^^-^^20^ Yang et al. reported that ABCB1 and ABCC3 polymorphisms were associated with response to chemotherapy and overall survival in osteosarcoma patients.^18^ Caronia et al. indicated that ABCB1 and ABCC3 polymorphisms may influence the efficacy of chemotherapy on clinical outcome of steosarcoma patients.^19^ Our study also reported similar results with previous ones, which indicated that ABCB1 polymorphism can have an important role in the interindividual differences in clinical outcome of osteosarcoma patients.

It is well known that ABCC3 is one kind of the multidrug resistance protein family (MRP), and it is expressed in various organs, such as liver, gallbladder and kidney.^21^^,^^22^ Previous studies have suggested that ABCC3 mRNA is associated with drug resistance, and several studies have reported an association between ABCC3 C-211T polymorphism and clinical outcome of cancer patients after receiving chemotherapy.^19^^,^^23^ However, we did not find a significant association between ABCC3 C-211T polymorphism and clinical outcome of osteosarcoma, which is difference from previous two studies.^18^^,^^19^ The discrepancy of the results may be explained by the differences in ethnicities, sample size and also by chance. Further studies are greatly needed to confirm the association between ABCC3 C-211T polymorphism and clinical outcome of osteosarcoma.

***Limitations of the Study:*** The current study has several limitations. First, the study was conducted in one hospital, and the sample could not better represent other populations. Second, number of cases is relatively small which may decrease the statistical power to find the differences between groups. Thirdly, multiple genes are involved in the clinical outcome of osteosarcoma, and thus other genetic factors should be considered in further studies.

## CONCLUSION

ABCB1 3435TT genotype and T allele may be associated with the clinical outcome of osteosarcoma patients in a Chinese population. ABCB1 C3435T polymorphism may be used as a genetic predicator of clinical outcome in osteosarcoma patients treated with chemotherapy.

## Authors Contributions:

*SXH, LAG, GXL & LY:* Designed and performed the study, did statistical analysis & editing of manuscript.

*SXH, ZHX & ZYL:* Did data collection and manuscript writing.
